# Endophytic *Epichloë* species and their grass hosts: from evolution to applications

**DOI:** 10.1007/s11103-015-0399-6

**Published:** 2015-11-05

**Authors:** Kari Saikkonen, Carolyn A. Young, Marjo Helander, Christopher L. Schardl

**Affiliations:** Management and Production of Renewable Resources, Natural Resources Institute Finland (Luke), Itäinen Pitkäkatu 3, 20520 Turku, Finland; The Samuel Roberts Noble Foundation, Ardmore, OK 73401 USA; Section of Ecology, Department of Biology, University of Turku, 20014 Turku, Finland; Department of Plant Pathology, University of Kentucky, Lexington, KY 40546-0312 USA

**Keywords:** Fungal endophytes, Grass, Genetic variation, Alkaloids, Coevolution

## Abstract

The closely linked fitness of the *Epichloë* symbiont and the host grass is presumed to align the coevolution of the species towards specialization and mutually beneficial cooperation. Ecological observations demonstrating that *Epichloë*-grass symbioses can modulate grassland ecosystems via both above- and belowground ecosystem processes support this. In many cases the detected ecological importance of *Epichloë* species is directly or indirectly linked to defensive mutualism attributable to alkaloids of fungal-origin. Now, modern genetic and molecular techniques enable the precise studies on evolutionary origin of endophytic *Epichloë* species, their coevolution with host grasses and identification the genetic variation that explains phenotypic diversity in ecologically relevant characteristics of *Epichloë*-grass associations. Here we briefly review the most recent findings in these areas of research using the present knowledge of the genetic variation that explains the biosynthetic pathways driving the diversity of alkaloids produced by the endophyte. These findings underscore the importance of genetic interplay between the fungus and the host in shaping their coevolution and ecological role in both natural grass ecosystems, and in the agricultural arena.

## Introduction

Specialization and coevolution have taken the center stage of discussion in evolutionary biology since Darwin emphasized in *Origin of Species* how species diversity and interactions together shape the evolution of life from individuals to communities (Darwin 1859; Thompson 1994). Now we know that virtually all species evolve in interactions with other species, interactive species often reciprocally affect each other’s evolution, and reciprocal changes in coevolving species often require and/or produce specialization. Thus, the majority of evolution fundamentally incorporates the elements of coevolutionary processes, and specialization commonly plays a role, especially in tightly linked species interactions such as symbiotic microbial interactions.

Interactions between endophytic *Epichloë* species and their host grasses provide a unique model for ecologists and evolutionary biologists interested in specialization in coevolving species interactions. By definition, fungal endophytes live internally and asymptomatically within organs of their host plant (Wilson 1995). These asymptomatic fungal infections are ubiquitous, abundant and taxonomically diverse residents in all terrestrial plants (Saikkonen et al. 1998; Rodriguez et al. 2009). The majority of endophytes are latent pathogens or dormant saprophytes and other fungal “hitch-hikers” lurking within the plant tissues without causing visible symptoms (Wilson 1995; Arnold et al. 2000; Saikkonen et al. 2004a, b; Saikkonen 2007; Rodriguez et al. 2009; Partida-Martinez and Heil 2011; Zabalgogeazcoa et al. 2013). In contrast to many other fungal taxa having asymptomatic endophytic periods in their life cycles, the endophytic *Epichloë* species (Leuchtmann et al. 2014) that are symbiotic with cool season grasses form systemic and life-long infections within their hosts. This extension of latency seen with *Epichloë* species is associated with reduction of virulence, adaptations and specialization that can promote fitness benefits to the host grass (Schardl 1996; Kover and Clay 1998; Saikkonen et al. 1998, 2004b, 2006, 2010a; Spatafora et al. 2007).

The symbiosis between *Epichloë* species and grasses is highly integrated involving the reciprocal use and manipulation of morphology, physiology, and life cycle and history traits of the partners to increase the fitness of the symbiota. First, the fungal hypha grows throughout the above-ground tissues of the host grass including inflorescences. It remains restricted to the intercellular spaces. Such an intimate relationship requires adaptations allowing the fungus to access the host plant interior, perhaps suppressing the recognition and defense responses that normally halt the establishment of harmful fungal infections in the host plant (Hamilton et al. 2012; Saikkonen et al. 2013a). The associated mechanisms are poorly understood but the oxidative balance is suggested to play a role (Hamilton et al. 2012). Second, the fitness of the partners is tightly linked, which should favor the evolution of interaction toward reduced antagonism and increased partner fidelity (Thompson 1994; Saikkonen et al. 2002).

*Epichloë* species are obligate associates of grasses subsisting entirely on the host grass. In addition to nutrient acquisition, grass reproduction provides a distribution avenue for the *Epichloë* species which are vertically transmitted in seeds from plant to its offspring. For strictly asexual *Epichloë* species vertical transmission is the only described means for distribution, whilst pleiotropic *Epichloë* species are capable of both vertical and horizontal transmission with asexual or sexual life cycles (Michalakis et al. 1992; Schardl 1996; Saikkonen et al. 1998; Tadych et al. 2012). At the other end of the continuum, truly sexual *Epichloë* species are horizontally transmitted by ascospores. Thus, the distribution of *Epichloë* species is largely determined by the fitness of the host particularly in the case of strictly asexual *Epichloë* species (but see Saikkonen et al. 2002). In exchange for hosting the endophyte, the host grass can receive benefits such as competitive superiority compared to uninfected counterparts in a population through increased growth and reproduction, as well as resistance to various abiotic and biotic stresses such as drought, flooding, pathogens and herbivores (Clay 1988, 2009; Saikkonen et al. 2006, 2010a; Song et al. 2015). Consequently, *Epichloë* species have the potential to markedly affect host fitness, exert strong selective pressure on grass host traits, and modulate grassland ecosystems (Clay and Holah 1999; Saikkonen 2000; Clay et al. 2004; Rudgers et al. 2004, 2007; Saikkonen et al. 2013a).

Similarly to other biological interactions based on mutual exploitation, benefits to *Epichloë* species and their host grasses are rarely symmetric. Thus, the symbiosis can range from antagonistic to mutualistic, and conflicting selection forces are likely to destabilize them. For example, when pleiotropic and antagonistic *Epichloë* species enter their sexual life cycle they produce external stromata surrounding some or all host inflorescences eliminating seed production. The benefits from endophytes appear to be dependent on the fungal and host genotype, and on environmental conditions. Accordingly, the symbioses are commonly regarded either as commensal or mutualistic. The major destabilizing forces in the symbiosis are asymmetry in dependence and genetic compatibility. Accumulating evidence has revealed that the grass does not necessarily depend on the fungus in some environments, many *Epichloë* strains are host species specific and genetic mismatch between host and symbiont can limit the endophyte-grass combinations (Saikkonen et al. 2004b, 2006, 2010b; Gundel et al. 2010, 2012, 2013).

In this paper we first dissect recent research advances and literature on endophytic *Epichloë* species, covering their evolutionary origin and taxonomical aspects, functional genetics, and coevolution with host grasses, and then examine their ecological roles and potential in novel solutions for sustainable agriculture. Accumulating findings have revealed that *Epichloë* species can reprogram host metabolism, and modulate photosynthesis, signaling and chemical cross-talk between the partners (Huitu et al. 2014; Eaton et al. 2010, 2015; Dupont et al. 2015) and thus, directly promote the growth, reproduction and competitive ability of the host grass (Clay and Holah 1999; Rudgers et al. 2004, 2007; Saikkonen et al. 2013b). However, here we focus on functional genetics driving alkaloid production because defense against herbivores is suggested to be the primary driving selective force behind the mutualism (Clay 2009; Saikkonen et al. 2010a).

## Speciation of endophytic *Epichloë* species and their cophylogeny with grasses

To understand the evolution of *Epichloë* species, it is necessary to consider the relationships of symbiont and host-plant life cycles—both sexual and asexual—and how those relate in turn to horizontal versus vertical transmission, and to haploid versus polyploid genomes.

The *Epichloë* species, as currently recognized by most, are systemic symbionts (or parasites) in the aerial parts of host plants in the C3 “cool season” grasses (Poaceae subfamily Pooideae), and either are choke pathogens or are related to choke pathogens (White 1993; Leuchtmann et al. 2014). Most *Epichloë* species can benignly colonize developing florets and seeds, facilitating efficient vertical transmission (Siegel et al. 1984; Tintjer et al. 2008). Horizontal transmission of some *Epichloë* species can occur either via asexual or sexual spores (Saikkonen et al. 2004a, b; Tadych et al. 2012). The choke pathogens can fruit on their hosts, forming a sporogenous stroma on the flag-leaf sheath and halting maturation of the subtending inflorescence (“choke” or “cattail” disease) (White 1997). The stroma produces spermatia and trichogynes (female receptive hyphae) and attracts female *Botanophila* sp. flies as “pollinators” that transfer spermatia. This results in cross-fertilization of the A and B mating types (MTs), which are determined by the *MTA* and *MTB* idiomorphs (alternative genes or gene clusters) at the *MT* locus (Schardl and Scott 2012; Schardl et al. 2014). The ensuing sexual stage generates haploid spores (“ascospores”) that can mediate horizontal transmission to developing seeds (Chung and Schardl 1997a) or growing plants (Meijer and Leuchtmann 1999).

In some hosts, sexual *Epichloë* species are observed only to transmit horizontally, but in most there can be a mixture of choked tillers and asymptomatic tillers, with the latter bearing the endophyte in the seeds (Sampson 1933; White 1994; Schardl 2001). Since there is no genetic difference in the fungus associated with choked versus asymptomatic tillers on an individual plant, it seems likely that this duality of reproductive processes has an epigenetic basis.

Most *Epichloë* species are incapable of fruiting on their hosts, and are therefore asexual and vertically transmitted. Some of these can still form sparse hyphal nets that produce some conidia (White et al. 1996), and theoretically could transmit horizontally as well, but vertical transmission seems by far the dominant process for asexual *Epichloë* species. (White et al. 1991; Moon et al. 2000, 2002; Chen et al. 2015) The majority of asexual *Epichloë* species are diploid or triploid interspecific hybrids possessing most or all of the genomes of two or three ancestral haploids, respectively. Such polyploid, asexual hybrids seem unusual among fungi, though well documented in the *Verticillium dahliae* species complex (Inderbitzin et al. 2011), and a common characteristic of parthenogenic lizards, fish, amphibians (Bogart et al. 2007; Lampert and Schartl 2010; Charney 2012) and nematodes (Lunt 2008). The lack of a vegetative incompatibility system in *Epichloë* species (Chung and Schardl 1997b), and presumably selection favoring some hybrids over ancestral haploids can account for the abundance of hybrid *Epichloë* species in nature (Faeth and Saari 2012).

**Fig. 1:**
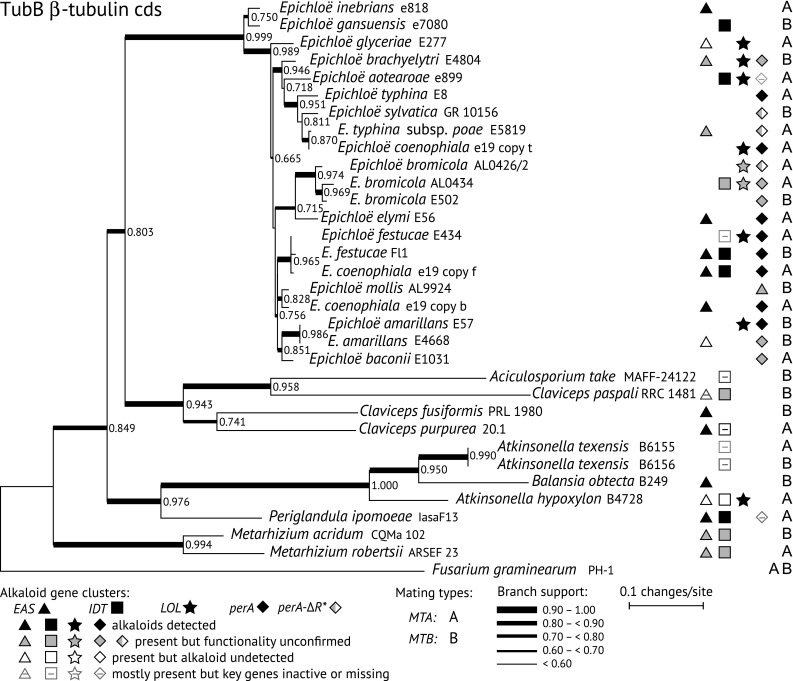
Phylogeny of TubB coding sequences (cds) for *Epichloë* species and related species. Gene coding sequences were identified by manual annotation of sequenced genomes. The tree was inferred by maximum likelihood search using PhyML without GBlocks curation. The tree was rooted with *Fusarium graminearum* PH-1 as the outgroup, and numbers on branches indicate ALR support. Alkaloid biosynthesis gene clusters and mating types are indicated after each strain designation, with symbols defined in the legends beneath the phylogram. Alkaloid gene clusters are for ergot alkaloids (*EAS*), indole-diterpenes (*IDT*) and lolines (*LOL*); and the multi-domain peramine synthetase gene (*perA*) and a related allele (*perA*-∆R*) are also indicated

Investigation of possible cophylogeny of haploid *Epichloë* species with their pooid grass hosts would suggest when this symbiotic system first emerged. The genetic analysis of *Epichloë* species and grasses indicated significant host-endophyte co-divergence (Schardl et al. 2008). Since then, an explosion of genome sequences for *Epichloë* species and related Clavicipitaceae has allowed more detailed phylogenetic analysis (Leuchtmann et al. 2014; Schardl et al. 2014; Chen et al. 2015). Strikingly, the deepest split identified is to a clade of two species symbiotic with *Achnatherum* species (Fig. 1) whose tribe (Stipeae) groups in a clade that splits early from most of the other pooid tribes from which *Epichloë* species have been sampled (GPWG 2001). Almost as basal is the branch to *E. glyceriae*, which is associated with another early diverging tribe (Meliceae). This contrasts with a clade in most housekeeping gene trees that includes *E. bromicola* and *E. elymi*, of which the former is found in members of sister tribes Hordeeae (=Triticeae) and Bromeae, and the latter is found just in Hordeeae. Similarly, another clade encompasses several *Epichloë* species that are found only in Poeae; namely, *E. amarillans, E. baconii, E. festucae, E. mollis* and *E. stromatolonga*. Cophylogeny is not consistently indicated for all species and clades (particularly not for the broad host range species *Epichloë typhina*), but evidence of a significant tendency for co-divergence suggests that the origin of genus *Epichloë* may have been close in time to the origin of the highly speciose grass subfamily, Pooideae (Schardl et al. 2008; Bouchenak-Khelladi et al. 2010; Ambrose et al. 2014).

Most of but not all asexual *Epichloë* species are interspecific hybrids (Moon et al. 2004; Charlton et al. 2012, 2014; Iannone et al. 2012; Oberhofer and Leuchtmann 2012; McCargo et al. 2014; Schardl et al. 2012). In hosts or regions where a single hybrid *Epichloë* species dominates, the implication is that the product of that hybridization was an endophyte that so enhanced its host’s fitness that it spread through much or all of its host’s range. An example is *Epichloë uncinata* (*E. bromicola* × *E. typhina* subsp. *poae*), which is found only in the grass *Lolium pratense* (=*Schedonorus pratensis* = *Festuca pratensis*; meadow fescue), and throughout the geographical range of its host (Ekanayake et al. 2012; Karimi et al. 2012). Only one isolate from this grass has been identified as a distinct *Epichloë* species, *E. siegelii* = *E. bromicola* × *E. festucae* (Craven et al. 2001). Likewise, the three-part hybrid, *Epichloë coenophiala*, dominates populations of *Lolium arundinaceum* (=*Schedonorus arundinaceus* = *Festuca arundinacea*; tall fescue) in northern Europe, central Asia (Ekanayake et al. 2012) and Iran (Karimi et al. 2012). On another continent, *E. tembladerae* has been found in numerous native grasses throughout Argentina (Iannone et al. 2012), including two species with a second hybrid endophyte; namely, *Bromus auleticus*, which can also host *Epichloë pampeana* (also *E. festucae* × *typhina* subsp. *poae*) (Iannone et al. 2009), and *Phleum alpinum*, which can also host *Epichloë cabralii* (*E. typhina* subsp. *poae* × a relative of *E. baconii*) (McCargo et al. 2014).

Although an increasing number of studies suggest context dependency of fitness benefits to the host grass from hybrid endophytes depending on e.g. environmental conditions and cascading trophic interactions, hybrid endophytes appear to increase host adaptability especially to extreme environments (Hamilton et al. 2009, 2010; Faeth and Saari 2012; Oberhofer et al. 2014; Saari et al. 2014).

One possible reason for interspecific hybrids to provide exceptional fitness contributions to host grasses is the production of anti-herbivore alkaloids. Both *E. coenophiala* and *E. uncinata,* and also *E. siegelii*, produce very high levels of loline alkaloids, which provide broad-spectrum protection from insects (Schardl et al. 2007). Most *E. coenophiala* strains also produce ergot alkaloids at levels that deter grazing by livestock, as well as the insect feeding deterrent, peramine (Christensen et al. 1993; Bush et al. 1997). Many of the endophytes in Argentina have genes for indole-diterpene biosynthesis, and this fits with symptoms suffered by livestock that ingest *Poa huecu*, which commonly hosts *E. tembladerae* (Cabral et al. 1999). Such poisonings are sometimes reported to be fatal to the animal. The other Argentine endophytes, *E. pampeana* and *E. cabralii*, also produce lolines (McCargo et al. 2014). The frequency of alkaloid genes appearing in hybrids is very high, suggesting that the alkaloids constitute a significant component of the fitness enhancement that is the basis for selection of the hybrid endophytes, as discussed in the following section.

## Genetic diversity of *Epichloë* species and alkaloid profile of symbiota

The bioactive alkaloids, ergot alkaloids, indole-diterpenes, lolines and peramine, can be produced by *Epichloë* species and likely provide selective advantages to the host species the endophytes inhabit. The lolines are strongly insecticidal and peramine acts as an insect feeding deterrent (Siegel et al. 1990; Riedell et al. 1991). The ergot alkaloids and indole-diterpenes are most well known for their toxicity to grazing livestock in the form of fescue toxicosis and ryegrass staggers, respectively, but can also exhibit anti-insect activity.

To understand alkaloid production initial research focused on identification of pathway end products for each alkaloid class. Genetics and molecular biology were used to identify genes encoding each pathway step and recombinant technology was used to dissect the biosynthetic pathways by gene knockouts, RNAi and heterologous gene expression (Panaccione et al. 2001; Spiering et al. 2002, 2005, 2008; Wang et al. 2004; Tanaka et al. 2005; Young et al. 2005, 2006; Saikia et al. 2012; Pan et al. 2014a, b). Apart from the *perA* gene that encodes peramine synthetase, the other alkaloid loci (*EAS* for ergot alkaloids, *IDT/LTM* for indole-diterpenes and *LOL* for lolines) are gene clusters that are often complicated by the presence of AT-rich repetitive sequences. Genome sequencing has enlightened us on the extensive genetic diversity of *Epichloë* species with respect to the known alkaloids and also provides information on other biosynthetic gene clusters, for many of which the products are yet to be elucidated (Schardl et al. 2013a, b, 2014).

Comparison of gene and genome sequences from species with differing alkaloid profiles has provided insight into the genetic variation that explains endophyte chemotypic diversity (Schardl et al. 2013a, b, 2014; Berry et al. 2015). Strains of *Epichloë* species that are unable to produce a specific alkaloid class are typically devoid of genes encoding key pathway steps. In many cases the whole genetic locus is absent, but sometimes remnant genes, pseudogenes or gene fragments from the locus can still be identified within the genome. Chemotypic diversity within a given pathway can also be identified. For example, the chemotypic difference between two *E. canadensis* strains symbiotic with *Elymus canadensis* that vary within ergot alkaloid (chanoclavine vs. ergovaline) and loline alkaloid (1-acetamidopyrrolizidine vs. *N*-acetylnorloline) pathway end products are explained by variation of the genes that are present (Charlton et al. 2012; Schardl et al. 2013b; Pan et al. 2014a, b). The *E. canadensis* isolate CWR5 has a functional *EAS* locus containing all 11 *EAS* genes enabling production of ergovaline and a *LOL* locus for production of *N*-acetylnorloline that lacks functional copies of *lolP, lolM* and *lolN*. (Note that isolates capable of producing *N*-formylloline would have functional copies of *lolP, lolM* and *lolN*). The *E. canadensis* isolate CWR34 lacks most *EAS* genes containing only functional copies of *dmaW*, *easF*, *easC* and *easE* encoding the steps for chanoclavine. The CWR34 *LOL* locus is similar to CWR5 except a small deletion in *lolO* renders the gene non-functional so the pathway stops earlier at 1-acetamidopyrrolizidine. Many other examples exist whereby the genetic variation between strains can explain differences in alkaloid chemotypes (Charlton et al. 2014; Takach and Young 2014; Young et al. 2014, 2015; Berry et al. 2015). In rare cases a whole gene cluster is present and contains no apparent deleterious mutations yet the corresponding alkaloid is not produced. It appears that these clusters are silent and gene expression is below a threshold level for functionality (Schardl et al. 2013b; Charlton et al. 2014).

Just as gene content can vary for an alkaloid locus, so can the gene arrangement within a locus. Many of the cluster rearrangements have likely occurred due to repetitive sequences within each locus. Interestingly the loci for ergot alkaloids and indole-diterpenes are located at a subterminal region of the chromosome, although the alkaloid genes located nearest the telomere can vary. The *EAS* clusters represent the greatest variation of cluster organization across *Epichloë* species with at least five different genes positioned nearest the telomere dependent on species or strain (Schardl et al. 2013a; Young et al. 2015).

**Fig. 2:**
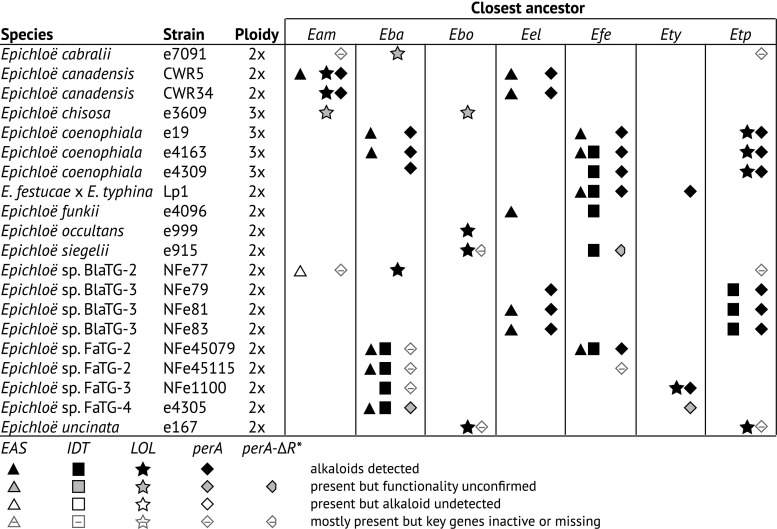
Ploidy of hybrid species and closest ancestor contributing alkaloid genes. The closest ancestors are indicated as *E. amarillans* (*Eam*), *E. baconii* (*Eba*), *E. bromicola* (*Ebo*), *E. elymi* (*Eel*), *E. festucae* (*Efe*), *E. typhina* (*Ety*) and *E. typhina* subsp. *poae* (*Etp*). Alkaloid gene clusters are for ergot alkaloids (*EAS*), indole-diterpenes (*IDT*) and lolines (*LOL*); and the multi-domain peramine synthetase gene (*perA*) and a related allele (*perA*-∆R*) are also indicated. Unnamed *Epichloë* taxa are abbreviated by host, *B. laevipes* Taxonomic Group (BlaTG-#) and *F. arundinacea* Taxonomic Group (FaTG-#)

The polyploid nature of the interspecific hybrid genome means that one or all ancestors can contribute alkaloid genes, which can allow for pyramiding of alkaloid classes (Fig. 2). Contributing ancestral species can be identified through phylogenetic analysis of the alkaloid genes and most often these are consistent with the species tree (Schardl et al. 2013b, Charlton et al. 2014, Berry et al. 2015). The alkaloid gene contributions in hybrid species are frequently found in the extant nonhybrid species. The chanoclavine genotype, *EAS*^*CC*^, is present in the hybrids *E. canadensis* (hybrid of *E. amarillans* × *E. elymi*) and *E. funkii* (*E. elymi* × *E. festucae*) and is contributed by *E. elymi*. There are other examples where the alkaloid gene contribution has only been found in the hybrid species and not in the extant ancestor. The *LOL* gene origin in *E. coenophiala* is *E. typhina* subsp. *poae*, but *LOL* genes are yet to be identified in this species (Kutil et al. 2007).

In addition to alkaloid diversity, some host species are able to form a symbiotic association with different endophyte species. Tall fescue, *Bromus laevipes*, *Elymus canadensis*, *Hordelymus europaeus*, *Achnatherum robustum* and *Achnatherum inebrians* can independently host more than one *Epichloë* species but this association is still limited to only one endophyte strain per individual plant (Christensen et al. 1993; Oberhofer and Leuchtmann 2012; Schardl et al. 2013b; Charlton et al. 2014; Takach and Young 2014; Chen et al. 2015; Shymanovich et al. 2015). The symbiont variation can be further expanded due to alkaloid chemotypic variation within a single species. Conversely, sometimes the same *Epichloë* species can be found in diverse host species. For example *E. tembladerae* is recognized as a symbiont of *Poa huecu*, *Festuca arizonica*, *Festuca argentina*, and *Bromus auleticus* (Cabral et al. 1999; Moon et al. 2004; Iannone et al. 2009) but it is unknown if chemotypic variation exists across this endophyte species.

The full extent of alkaloid diversity associated with *Epichloë* species is only now being realized as our ability to genetically evaluate the endophyte directly within the plant has improved (Charlton et al. 2014; Takach and Young 2014; Young et al. 2014, 2015; Chen et al. 2015; Shymanovich et al. 2015). How this endophyte diversity is translated to host fitness enhancement needs to be further explored.

## Ecological consequences of genetics in nature and man-made environments

The growing literature illustrates the importance of genetics for symbiotic *Epichloë* species and their host grasses in both evolutionary and ecological time-scales. Phylogenetic analyses suggest the co-origin of genus *Epichloë* and the grass family Pooideae (Schardl et al. 2008) explaining high prevalence of *Epichloë* species in this particular grass family. Co-phylogeny is not consistent for all species and clades but in co-diverged phylogenetic branches hybridization is commonly detected (Moon et al. 2004; Charlton et al. 2012; Iannone et al. 2012; Oberhofer and Leuchtmann 2012; McCargo et al. 2014). Genetic compatibility between the fungal strain and the host lineage appears to play significant role in establishment of endophyte-grass combinations, and transgenerational maternal effects can affect the genetic structure of a host population (Saikkonen et al. 2010b). Genetic variation between *Epichloë* strains explains differences in alkaloid chemotypes (Charlton et al. 2014; Takach and Young 2014; Young et al. 2014, 2015; Berry et al. 2015) and the frequency of alkaloid genes is high in hybrids allowing for pyramiding of alkaloid classes. Thus, hybridization can result in significant fitness enhancement and selective advantage to hybrid endophytes. However, genetic differences among fungal lineages fail to explain for example, the duality of reproductive sexual and asexual strategies of the fungi. These observations suggest that specialization and genetic interplay between the endophyte and the host grass can largely explain phenotypic variation in the symbiotum and its ecological consequences but also suggests that other mechanisms such as phenotypic plasticity and epigenetic modifications in gene expression and function are likely to play a significant role in ecologically relevant traits of the *Epichloë*-grass symbiosis.

In nature, endophytic *Epichloë* species can affect the host growth and reproduction, the structure of grassland communities and trophic interactions, and thereby adaptive radiation of *Epichloë* species and their host grasses (see e.g. Clay and Schardl 2002; Clay et al. 2004; Rudgers et al. 2004, 2007; Saikkonen et al. 2004a, 2006, 2010a; Rodriguez et al. 2009). Because grasses dominate approximately 40 % of the Earth’s surface, *Epichloë* species are likely to have significant ecosystem consequences as well.

The potential applications are related to successful grass production management in the changing climate. For example, economical value of systemic grass-endophytes related to forage quality and biocontrol has already been widely recognized in agriculture and turf grass industry in the USA and New Zealand (Hoveland 1993; Gundel et al. 2013; Johnson et al. 2013). Economic losses caused by poor animal performance feeding on endophyte infected forage of tall fescue and perennial ryegrass in the United States only have been estimated at $600 million annually (Hoveland 1993). On the other hand, fungal strains which do not produce mycotoxins harmful to cattle but increase biomass production, seed production and germination, stress tolerance (e.g. drought, flooding, temperature, and pest, pathogen and weed invasions), silicon, secondary metabolite or nutrient content should be taken into account when aiming to increase forage productivity when introduced to forage cultivars (Clay and Schardl 2002; Lehtonen et al. 2006; Saikkonen et al. 2013b; Vázquez-de-Aldana et al. 2013; Huitu et al. 2014; Song et al. 2015). One of the most successful commercial example of such an animal-safe non-toxic endophyte is ‘‘MaxQ’’ (*E. coenophiala*) in the tall fescue variety “Jesup” (Johnson et al. 2013). Examples of commercially successful novel endophytes providing bio-protective properties to the host plant against insect pests are e.g. “AR1, AR5, AR37 and NEA2” endophyte strains which have been selected and transferred to perennial ryegrass cultivars (Johnson et al. 2013). Different endophyte strains, however, exhibit remarkable variation in alkaloid types, and levels of alkaloids are context dependent (Bony et al. 2001; Johnson et al. 2013). The positive effects of endophytes appear to be more pronounced in nutrient-rich environments. Recent evidence suggests that also warming and drought stress can affect the alkaloid production of endophytes (Hill et al. 1996; Brosi et al. 2009; Compant et al. 2010) suggesting that defensive mutualism should be taken into account in grass production management in the changing climate. The associated economic and food safety profits of using these endophyte improved grass cultivars include lower investments in chemical pest control when using natural biocontrol and consumers avoid remnants of chemical pesticides in the crop, meat and milk.

Current biotechnological knowledge allows us to utilize endophytic *Epichloë* species in agribusiness (Gundel et al. 2013; Johnson et al. 2013). Endophytic fungi can be routinely eliminated from host plant seeds by heat treatments or fungicides and new strains introduced into the uninfected plants by inoculating the hyphae into the plant tissue. Furthermore, recent discoveries in genome mapping techniques allow identification and location of genes that encode the information that have ecological importance. In addition to enhanced mycotoxin production we are only beginning to understand genetic bases of other adaptive fungal and grass traits. This knowledge and the tools of contemporary genetics widen the possibilities of plant breeding from utilization of selected endophyte-plant manipulations to the transfer of gene(s) from the fungus to grasses or other crop plants such as cereals. Thorough understanding of mechanisms underlying variation, heritability and stability of cultivar traits are, however, required to understand responses of grass cultivars to environmental change and their successful use in different environments.

## Conclusions and future perspectives

The importance of endophytic *Epichloë* species to focal ecosystem functions driving both below- and aboveground food webs is well recognized and accepted (Omacini et al. 2001; Clay and Schardl 2002; Clay et al. 2004; Rudgers et al. 2004; Saikkonen et al. 2006, 2010a, 2013a, b, 2015; Rudgers et al. 2007; Omacini et al. 2012). Recent phylogenetic and molecular analyses coupled with accumulating ecological approaches have provided insights into the coevolution of *Epichloë*-grass symbiosis and how genetic interplay between the partners can have great repercussions also in a ecological time-scale. Reproduction and transmission mode (vertical vs. horizontal) of *Epichloë* species as well as architecture and lifespan of the host grass are important factors related to the epidemiology, genetic compatibility, specialization and evolution of avirulence in *Epichloë* species. However, the general questions to be solved in future studies are (a) what is the relative importance of phenotypic plasticity and heritable (genetic and/or epigenetic) variation in ecologically relevant grass traits, (b) how selection operates on the unitary, modular or super organism levels of *Epichloë*-grass associations, (c) how the phenotypic unit of the symbiotum mediates plant–plant and trophic interactions in grassland communities, and (d) species distribution ranges. Until now the lack of this knowledge has limited the use of full potential of endophytic *Epichloë* species in sustainable agriculture.
